# Pulmonary Alveolar Proteinosis Refractory to Plasmapheresis and Rituximab despite GM-CSF Antibody Reduction

**DOI:** 10.1155/2022/2104270

**Published:** 2022-01-30

**Authors:** Aysenur Keske, Eric M. Destrampe, Byron Barksdale, William N. Rose

**Affiliations:** Department of Pathology, University of Wisconsin Hospital, 600 Highland Ave, Madison, WI 53792, USA

## Abstract

We share our experience of a patient with pulmonary alveolar proteinosis who was refractory to plasmapheresis and rituximab despite a significant reduction in the offending antibody. He presented with shortness of breath, fevers, chills, and sweats for 4 months. He was diagnosed with autoimmune PAP based on typical radiology findings, bronchoalveolar fluid analysis, and elevated anti-GM-CSF levels. Given his limited improvement with whole lung lavage and inhaled GM-CSF therapy, he underwent two series of plasmapheresis. Series one was 5 procedures in 6 days, and series two was 5 procedures in 9 days followed by rituximab. These did not appear to provide any lasting clinical benefit in the year after plasmapheresis despite a marked decrease in serum anti-GM-CSF levels. However, about a year after plasmapheresis, he went into remission and has not required any treatment.

## 1. Introduction

Pulmonary alveolar proteinosis (PAP) is a rare disorder that is due to disrupted surfactant production or macrophage-mediated clearance that leads to alveolar surfactant accumulation and an impairment of gas exchange [1]. The disease can present with various manifestations ranging from exertional dyspnea to superimposed life-threatening opportunistic infections and hypoxic respiratory failure [1].

PAP is heterogeneous, as it can have multiple etiologies. Primary PAP is subdivided into hereditary or autoimmune (previously known as idiopathic or acquired) forms that are associated with mutations of genes regulating surfactant metabolism (such as CSF2RA) and autoantibodies against GM-CSF, respectively [1]. Hematologic disorders and environmental exposures (such as silica) may impair macrophage function and cause secondary PAP [2, 3]. Autoimmune mechanisms account for more than 90% of the cases [4]. Despite the known relationship between GM-CSF autoantibody-mediated impairments in macrophage function and surfactant accumulation in alveolar spaces, no correlation between circulating autoantibody levels and disease severity has been reported [4–6]. However, bronchoalveolar fluid (BALF) autoantibody levels appear to correlate with markers of disease severity (such as radiological involvement of lung, AaPO2, PaO2, and serum LDH levels) [5].

Whole lung lavage (WLL) is often described as the standard of care and commonly used for rapid symptom relief within days for symptomatic patients. A very rough estimate of “the response rate” is 60% [7]. In the case of refractory or worsening symptoms, inhaled GM-CSF therapy can be used. Plasmapheresis and rituximab are rarely used therapies, as only few case reports have examined the effectiveness of these therapies. To add our experience to this sparse literature, we present a case report of a patient with autoimmune PAP who did not show improvement following two series of plasmapheresis.

## 2. Case Presentation

The patient is a 28-year-old male with a history of asthma and smoking. He presented to an outside hospital with progressive shortness of breath, fevers, chills, and sweats for 4 months. A computed tomography (CT) scan demonstrated bilateral geographic distributions of ground glass opacities with interspersed interlobular septal thickening.

He described a productive cough with clear-to-white phlegm. He took a short course of amoxicillin-clavulanate without improvement in his symptoms. His shortness of breath progressed significantly after 4 months, and he also developed hemoptysis. He was admitted to an outside hospital where his initial CT chest showed multifocal areas of ground glass opacity in the upper and lower lobes with relative sparing of the periphery.

The differential diagnosis at that time included pneumocystis pneumonia, eosinophilic pneumonia, and organizing pneumonia, vasculitis, autoimmune diseases, and hypersensitivity pneumonitis. No organic antigen exposure was identified in his history. Infectious disease markers for respiratory viruses, HIV, mycobacteria, and fungi were negative. Autoimmune and inflammatory markers (including ESR, CRP, ANA, ANCA, RF, and complement levels) were negative.

Bronchoscopy with BAL was performed and revealed a milky fluid that was PAS positive. He was discharged for outpatient follow-up. He reported hypoxia at home (saturations usually around mid to lower 80 s) with any activity.

He was unable to tolerate PFT on his outpatient visit. His SPO2 on room air was 88%, and he required 2 liters per minute (LPM) to keep his SpO2 greater than 89%. Given his persistent symptoms, he was referred to our hospital for initiation of WLL.

He reported a consistent cough with exertion and clear phlegm during his initial evaluation at our hospital. He denied sick contacts or recent travel. He was working at a foundry making silica sand into casts for the past 3 years (6 days a week and 10 hours a day). He did not report history/symptoms of GERD. No family history of PAP was reported. He did not take any medications other than as needed ibuprofen. Of note, he had no history of statin use. He was an active smoker with a 7 pack-year history. Occult hematologic malignancy as a cause of secondary PAP was excluded by flow analysis. Given his high antibody levels, autoimmune PAP was favored but PAP secondary to silica exposure could not be excluded.

The first bilateral WLL was performed, and he was successfully extubated following a brief course of mechanical ventilation. He did not require O_2_ at rest, but did require up to 2 L of O_2_ with activity when he was discharged. He reported initial improvement in his respiratory status that lasted a couple of days. He had increasing exercise intolerance, increasing production of purulent sputum, and intermittent chills and sweats 5 days after the procedure.

A repeat CT chest performed at an outside hospital again showed a “crazy-paving” pattern with areas of subpleural sparing. Slight temporal progression within the basilar segments of the right and left lower lobes were reported. He required 2 L of oxygen at rest and 2–4 L on exertion with an SpO2 of 72% on room air. He underwent a repeat WLL 20 days after the initial procedure. He was mobilized shortly after the procedure and discharged on the following day.

Two weeks after the second WLL, he reported worsening dyspnea and hypoxemia for a few days. He also reported increasing production of purulent sputum and intermittent chills and fevers. He denied any chest pain, hemoptysis, orthopnea, edema, or weight gain. His measured saturations were in the 70 s on room air and mid-90 s on 3 L of O_2_ at the outside outpatient clinic. His CXR showed worsening bilateral opacities (more prominent on the left) when compared to before, concerning for exacerbation of his PAP. His arterial blood gas analysis showed the following values on 2 L O_2_ : PH = 7.45, PCO2 = 27, PO2 = 73, BICARBONATE = 18.0, BASE EXCESS = −4.0, and O_2_ SATURATION = 94.1. Of note, no posttreatment arterial blood gas was available. His ambulatory oximetry study demonstrated a gradually increased oxygen requirement up to a maximum of 5 L.

Given the recurrent symptoms after WLL #2, he was admitted for WLL #3 as well as a series of 5 plasmapheresis procedures in 6 days. Each therapeutic plasma exchange (TPE) consisted of a 1-plasma volume exchange via centrifugal apheresis with 5% albumin as the replacement fluid.

His measured anti-GM-CSF autoantibody level was 103 mcg/mL (normal <5.0) prior to the first TPE. After the third plasmapheresis procedure, his anti-GM-CSF level decreased to 17.6 mcg/mL, but the patient reported no significant clinical improvement.

He was started on inhaled GM-CSF at the conclusion of this series of 5 TPEs. Just prior to being discharged, his ambulatory oximetry study demonstrated a minimally increased oxygen requirement up to a maximum of 1 L. He was kept on nebulized GM-CSF and repeated WLLs every 3-4 weeks due to refractory disease for a duration of about four months.

Four months after the first TPE series, another TPE series was tried. Despite treatment with WLLs in the intervening period, he did not see any lasting improvement. Thus, TPE was attempted again because it was well tolerated and has a relatively low side-effect profile. He received a second series of 5 TPEs in 9 days. He was also given rituximab (1000 mg IV) once after the last procedure. Unfortunately, he reported no significant clinical improvement after the second TPE series.

For about one year after the second TPE series, he continued to require WLLs every 3-4 weeks despite continuous inhaled GM-CSF treatment. At that point, he reported that he felt better. For 3 months, he continued the GM-CSF treatment but did not undergo any WLLs. Also, at that point, he also stopped GM-CSF. About 3 months after stopping GM-CSF, another 6-minute walk test was performed. He did not require any supplemental oxygen either at rest or after walking about 1300 feet. The most recent plan was to continue to hold both GM-CSF and WLL, as he is in remission.

## 3. Discussion

As of this writing, we were able to identify only four case reports that were published in English in which plasmapheresis was used for the treatment of autoimmune PAP due to incomplete responses to WLL. The first case demonstrated a marked reduction in serum antibody level (1 : 6400 to 1 : 400) and simultaneous clinical improvement following 10 TPEs [8]. The authors concluded that circulating antibody levels are likely to correlate with disease activity and can be used to measure treatment response.

However, no clinical improvement was reported in another patient who was treated with the same treatment schedule despite a reduction in serum antibody level (250 *μ*g/mL to 156 *μ*g/mL) [9]. Only short-term improvement was reported in a third patient who was treated with bilateral WLLs followed by 5 TPEs in 2 weeks [10].

A case of five consecutive daily TPEs followed by rituximab was also reported [11]. A significant reduction in anti-GM-CSF levels (24.8 to 2.7 *μ*g/mL) and a relatively long-term symptomatic remission were achieved by this protocol. This suggested a possible benefit of a more intense plasmapheresis regimen along with rituximab. However, a similar plasmapheresis protocol followed by rituximab did not provide a clear benefit in our patient despite achieving a marked reduction in the offending antibody level.

A marked decrease in serum antibody levels with a more intense plasmapheresis protocol is an expected result given the fact that TPE decreases IgG [12]. In our patient, his antibody level decreased from 103 mcg/mL just before the first TPE to 17.6 mcg/mL between the third and fourth TPEs. When accounting for reequilibration, the antibody level between TPE 3 and 4 can be estimated to be about 65–80% of the starting level [12]. This roughly corresponds to what was measured in our patient, as his antibody decreased by about 80% from pre-TPE 1 to post-TPE 3.

However, we speculate that the absence of clinical improvement for our patient during the year after two series of TPE may suggest a weaker correlation between the circulating antibody level and disease activity. Moreover, the timing of his remission long after TPE does not support a temporal relationship between TPE and clinical improvement.

Interestingly, BALF autoantibody levels were not obtained in any of the previous patients including our case, although previous studies reported conflicting results regarding the correlation between serum antibody levels and disease activity. Serum antibody levels are required for making a diagnosis of autoimmune PAP confidently as well as ruling out secondary PAP (along with additional clinical data). We speculate that BALF antibody levels may correlate more strongly.

Some discussion of the soundness of the diagnosis is warranted. The diagnosis of autoimmune PAP is mainly based on BALF findings and serum GM-CSF autoantibody levels [7]. Our patient was diagnosed with autoimmune PAP at an outside hospital where BALF results were consistent with PAP and a high serum antibody level was also found. These results were verbally reported to the clinical team at our institution. A serum antibody test was also ordered by our clinical team and confirmed the serologic diagnosis of autoimmune PAP prior to the first TPE.

At the time of diagnosis, the patient was working in a foundry where he had been exposed to silica and sand for 2.5 years. He was an active smoker with a 7 pack-year smoking history.

Since GM-CSF autoantibody levels are not elevated in secondary PAP, the diagnosis of autoimmune PAP was made after excluding other causes. His exposures cannot be ruled out as contributors, but they were deemed less likely due to the short durations. An association between silica exposure and autoimmunity has been reported [13]. Thus, it is possible that a persisting silica-mediate autoimmune disease could be a factor in his initial poor response to treatment. He left his job in the foundry approximately 6 months after initial diagnosis and cut down on his smoking. Those interventions may contribute his clinical improvement which may also support the possible effect of environmental exposure on the development of autoimmune PAP. However, silica-mediated immunological alterations are not well understood, and we do not have enough information on this patient to come to a definitive conclusion.

Additionally, treatment response in our case was mainly evaluated based on the patient's subjective symptoms, pulse oximeter readings as reported by the patient, and the more objective PaO2 values that were measured during hospitalization. A chest X-ray was usually obtained both on admission and following WLL. A pulmonary function test (PFT) performed about 12 months after initial diagnosis and 11 after the first series of TPE at our hospital was generally within normal limits (FEV1: 3.61 L, FEV1%PRED: 84, FVC: 4.33 L, FVC% PRED: 84, and FEV1/FVC: 0.83) except for DLCO (uncorrected for hemoglobin) which was very mildly reduced at 20.87 ml/min/mmHg (67% of predicted). He was unable to tolerate a pre-TPE PFT. LDH levels were not obtained. No other ambulatory oximetry study results or other functional test results are available other than the ones we have included. We speculate that more objective data could have provided additional information about the patient's status following plasmapheresis. However, these all have costs and limitations, and the ultimate “test” (the patient's self-reported well-being) consistently yielded disappointing results.

## 4. Conclusions

We share our experience of a patient with refractory autoimmune PAP who was treated with 2 series of plasmapheresis as well as rituximab but did not improve clinically despite a marked reduction in serum anti-GM-CSF level. The case is unusual because of his poor response to therapy. While we can only speculate about why his response to treatment was so poor, we hope that sharing our experience can contribute to a better understanding of autoimmune PAP and related conditions. There was a very long interval between antibody reduction and remission, as he went into remission about a year after plasmapheresis. This suggests that the antibody reduction therapies were potentially not the imputable cause of his remission.

## Data Availability

No data were used to support the findings of this study.

## Abbreviations

PAP: Pulmonary alveolar proteinosis
GM-CSF: Granulocyte-macrophage colony-stimulating factor
BAL(F): Bronchoalveolar lavage (fluid)
AaPO2: Alveolar-arterial oxygen pressure difference
PaO2: Partial pressure of oxygen
SpO2: Oxygen saturation
TPE: Total plasma exchange
LDH: Lactate dehydrogenase
CT: Computed tomography
WLL: Whole lung lavage
L: Liter
*μ*g/mL: Microgram/milliliter
Ig G: Immunoglobulin G
PFT: Pulmonary function test
FEV1 (%PRED): Forced expiratory volume (% predicted)
FVC (% PRED): Forced vital capacity (% predicted)
DLCO: Diffusing capacity for carbon monoxide
ESR: Erythrocyte sedimentation rate
CRP: C-reactive protein
ANA: Antinuclear antibody
ANCA: Antineutrophil autoantibody
RF: Rheumatoid factor.
